# Response-guided bulevirtide ± pegylated interferon alfa-2a: Long-term outcomes observed in the nationwide Austrian hepatitis D cohort study

**DOI:** 10.1016/j.jhepr.2026.101835

**Published:** 2026-03-26

**Authors:** Michael Schwarz, Marlene Hintersteininger, Caroline Schwarz, Marlene Panzer, Nikolaus Pfisterer, Nina Loschko, Lukas Hartl, Livia Dorn, Hermann Laferl, Michael Trauner, Albert F. Stättermayer, Mattias Mandorfer, Ivo Graziadei, Andreas Maieron, Alexander Moschen, Elmar Aigner, Vanessa Stadlbauer, Christian Madl, Stephan W. Aberle, Heinz Zoller, Michael Gschwantler, Thomas Reiberger, Mathias Jachs

**Affiliations:** 1 Division of Gastroenterology and Hepatology, Department of Medicine III, Medical University of Vienna, Vienna, Austria; 2 Department of Internal Medicine 2, Gastroenterology and Hepatology, University Hospital of St. Pölten, Karl Landsteiner University of Health Sciences, St Pölten, Austria; 3 Department of Internal Medicine IV, Klinik Ottakring, Vienna, Austria; 4 Department of Internal Medicine I, Medical University of Innsbruck, Innsbruck, Austria; 5 Department of Internal Medicine IV, Klinik Landstraße, Vienna, Austria; 6 Department of Internal Medicine 2, Johannes Kepler University Hospital Linz, Linz, Austria; 7 Department of Internal Medicine IV, Klinik Favoriten, Vienna, Austria; 8 Department of Internal Medicine, Academic Teaching Hospital Hall, Hall in Tirol, Austria; 9 First Department of Medicine, Paracelsus Medical University, Salzburg, Austria; 10 Division of Gastroenterology and Hepatology, Department of Internal Medicine, Medical University of Graz, Graz, Austria; 11 Center for Virology, Medical University of Vienna, Vienna, Austria; 12 Sigmund Freud University, Vienna, Austria

**Keywords:** Hepatitis D, Bulevirtide, Interferon, Pegylated interferon, PegIFN, ACLD, Response-guided therapy, HDV, Viral hepatitis, Treatment discontinuation

## Abstract

**Background & Aims:**

Chronic hepatitis D (CHD) often progresses to advanced chronic liver disease (ACLD). Bulevirtide (BLV) is approved for CHD, yet treatment duration, management of suboptimal response, and the potential for finite treatment remain unclear.

**Methods:**

Patients receiving BLV at 10 Austrian centers were included. Virological, biochemical, and combined response (VR/BR/CR) were assessed every 6 months (M6-M24). Pegylated interferon alfa-2a (PegIFN) was offered to suboptimal responders.

**Results:**

Sixty-one patients (median age: 45 years, 60.7% men, ACLD: 68.9%) receiving BLV for a median of 29.0 months were included. VR (Month [M]6: 36.4%, M12: 64.2%, M24: 61.9%), BR (M6: 56.4%, M12: 69.8%, M24: 66.7%), and CR (M6: 25.5%, M12: 47.2%, M24: 42.9%) were maintained for 2 years. Liver stiffness and systemic inflammation (*i.e.* C-reactive protein [CRP] and procalcitonin [PCT]) decreased under BLV treatment (all *p* <0.01). Nineteen patients (31.1%) received add-on PegIFN to BLV monotherapy after a median of 10.5 months, inducing a further HDV-RNA decline by 1.65 (IQR 0.81–2.11) log_10_ copies/ml and reductions in HBsAg levels by 0.08 (IQR 0.02–0.12) log_10_ IU/L after 24 weeks of combined therapy (both *p* <0.01). Overall, 32.8% (20/61 patients) achieved HDV-RNA target not detected (TND). Ten (seven BLV mono and three BLV + PegIFN) stopped treatment after 23.0 (IQR 12.0–29.0) months. Seven patients maintained HDV-RNA TND through the last follow-up (median 36.0 months), whereas three patients relapsed but achieved TND again following BLV retreatment.

**Conclusions:**

High response rates to BLV were observed in this nationwide cohort. In suboptimal BLV responders, PegIFN add-on was associated with a significant and partly sustained decline in HDV-RNA and HBsAg, indicating a relevant contribution to long-term viral infection control. Sustained negative HDV-RNA could help identify candidates for finite BLV treatment.

**Impact and implications:**

CHD is a severe form of viral hepatitis with rapid progression to cirrhosis and hepatocellular carcinoma, highlighting the need for effective treatments. In this real-world cohort of 61 Austrian patients, BLV significantly reduced HDV-RNA, alanine aminotransferase, and liver stiffness (*p* <0.001). Add-on PegIFN resulted in a further decline of HDV-RNA and HBsAg by 24 weeks of combined treatment (*p* <0.01) in 19 patients with suboptimal response to BLV treatment, and long-term HDV-RNA TND allowed elective treatment discontinuation in 10 patients under close surveillance.

## Highlights

• High BLV response rates were observed in a nationwide Austrian HDV cohort.
• PegIFN add-on yields further HDV-RNA and HBsAg declines in suboptimal responders.
• Sustained TND could help identify candidates for finite BLV therapy.

## Graphical abstract

**Figure:**
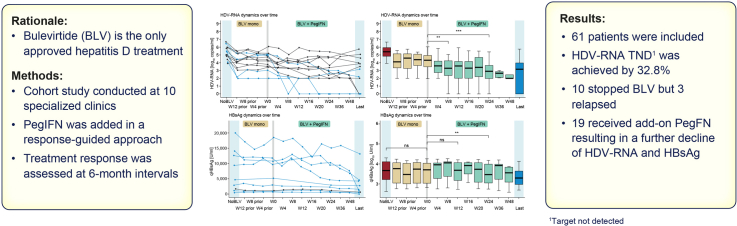

## Introduction

Chronic hepatitis D (CHD) is a severe form of viral hepatitis.^1^ The hepatitis D virus (HDV) is an incomplete virion that requires concurrent infection with HBV because it relies on the HBsAg protein to assemble its viral envelope.^2^^,^^3^ Globally, ∼250 million people live with chronic HBV (CHB) and it is estimated that 2–13% are co-infected with HDV, corresponding to ∼12 million cases of CHD.^4^^,^^5^ Compared with HBV mono-infection, patients with CHD are at an increased risk of developing hepatocellular carcinoma (HCC) or hepatic decompensation (*i.e.* ascites, variceal bleeding, or hepatic encephalopathy).^4^^,^[6], [7], [8], [9]

For many years, off-label pegylated interferon (PegIFN) was the only recommended treatment for CHD and achieved moderate response rates.[10], [11], [12] In 2020, the sodium taurocholate co-transporting polypeptide (NTCP) inhibitor bulevirtide (BLV), an entry inhibitor, was approved for the treatment of CHD.^13^^,^^14^ BLV treatment is associated with high safety and efficacy and has been recommended by international guidelines for the treatment of CHD.^15^ Based on seminal studies, BLV can be used either as monotherapy or in combination with PegIFN, which could enhance response rates while impairing the tolerability of treatment.^16^ However, the ideal treatment regimen and duration need to be defined. In Austria, there is a response-guided step-up approach for patients with CHD: PegIFN is offered as add-on treatment to patients who fail to respond to monotherapy, or if a plateau in HDV-RNA under BLV monotherapy occurs that renders the achievement of the goal of treatment (*i.e.* reaching virological response) unlikely.^17^

In this study, we report pretreatment characteristics and long-term outcomes of patients receiving BLV treatment at 10 participating viral hepatitis clinics in Austria. The aim of this nationwide cooperation was to assess long-term treatment efficacy and safety, further characterize the effects of add-on PegIFN treatment, investigate effects of treatment on markers of liver disease and systemic inflammation, to potentially identify predictors of treatment response, and to assess the possibility of a finite treatment duration.

## Materials and methods

### Study cohort

Stable outpatients with CHD who initiated BLV treatment at one of the 10 participating centers (Medical University of Vienna, Klinik Ottakring, Klinik Favoriten, Klinik Landstraße, Medical University of Innsbruck, Hospital Hall in Tirol, Keppler Medical University Linz, Medical University of Salzburg, Medical University of Graz, University Hospital of Sankt Pölten) from 2019 onwards were included. The methods are additionally provided in the Supplementary CTAT table.

### Virological, biochemical, and liver disease severity assessment

Assessed data included virological, laboratory, and histological reports. Laboratory data were used to calculate the Model of End-stage Liver Disease (MELD) score, aspartate aminotransferase (AST) to platelet ratio index (APRI), and Fibrosis-4 (FIB-4) scores, and, when available, enhanced liver fibrosis (ELF) test. Quantitative HDV-RNA PCRs were performed by the Center for Virology of the Medical University of Vienna using an in-house assay developed with external reference,^18^ with a limit of detection of 100 copies/ml. The RoboGene HDV Quantification Kit 2.0 (Roboscreen Diagnostics, Leipzig, Germany) with a lower limit of quantification of 8 IU/ml was used at the Medical University of Innsbruck and Hospital Hall. Thus, a conversion factor of 37 was used to ensure comparability.^17^ Liver stiffness measurements (LSMs) were conducted by using Fibroscan (Echosens, France, varying models at respective centers). The level of steatosis according to a controlled attenuation parameter (CAP) was defined as: S0, 150–247 dB/m; S1, 248–268 dB/m; S2, 268–279 dB/m; and S3, ≥280 dB/m.^19^ Advanced chronic liver disease (ACLD) was defined as a compound variable including at least one of the following parameters: LSM ≥10 kPa, liver biopsy histology of F3/F4, and hepatic venous pressure gradient measurement of ≥6 mmHg. Significant alcohol intake was defined as self-reported consumption of ≥20 g/day.^20^

### Definition of treatment response and add-on PegIFN treatment

The baseline (BL) was defined as the last visit before the first subcutaneous administration of BLV. Outcome data are presented in 6-month intervals (M6, M12, M18, and M24) in this report. Response criteria were defined in accordance with clinical trials: virological response (VR) defined as an HDV-RNA decline of ≥2 log_10_ units or HDV-RNA target not detectable (TND); biochemical response (BR) defined as normalization of alanine aminotransferase (ALT; ≤35 U/L for women and ≤50 U/L for men), and combined response (CR; defined as VR plus BR).^21^ Patients with an HDV-RNA decline of <2 log_10_ from baseline were classified as having a suboptimal response. Sustained HDV-RNA TND was defined as serum HDV-RNA levels below the lower limit of detection continuously for at least 24 weeks. If patients reached sustained HDV-RNA TND, treatment discontinuation was offered. After treatment discontinuation, patients were closely monitored at their respective outpatient clinic. Patients in whom BLV was discontinued following viral suppression who did not relapse were carried on as CR in further analyses. Patients who permanently discontinued BLV for other reasons (liver transplantation, death, etc.) were censored from further endpoint analysis (BR, VR, or CR) at the time of treatment discontinuation. Incidental HCC, liver transplantation, and death were recorded during treatment.

According to the Austrian response-guided treatment approach for patients with CHD, add-on PegIFN was considered once a virological plateau (*i.e.* no further decreases in HDV-RNA) was reached, irrespective of whether VR had previously been achieved.^17^ Furthermore, PegIFN was evaluated in patients without VR (*i.e.* a <2 log_10_ decline and still detectable HDV-RNA) after 6–12 months of BLV treatment. Add-on PegIFN treatment was offered to patients by the respective attending physician and the decision to initiate PegIFN was ultimately made on a case-by-case basis, considering contraindications to PegIFN and patient preference. The standard dose of PegIFN was 180 μg per week s.c.; however, reduced doses were administered in patients with impaired liver function, poor tolerability with chronic fatigue or flu-like symptoms, cytopenia (anemia, thrombopenia, or leukopenia), or other clinical considerations, such as patient preference. The planned treatment duration for add-on PegIFN was 48 weeks but could be extended at the discretion of the treating physician based on the course of HDV-RNA, HBsAg levels, and tolerability. In patients treated with add-on PegIFN, HDV-RNA, ALT, and HBsAg levels were assessed in 4-week intervals.

### Statistical analysis

For data curation, Microsoft Excel was used (Office 2019, Microsoft, Redmond, WA, USA). For statistical analysis and data visualization, Rstudio (Build 764, Posit Software, Boston, MA, USA) was used. Continuous variables were presented as median (and IQR displayed by first [Q1] and third [Q3] quartile) or as absolutes (n, and %). For statistical comparison of categorical variables, Chi-square test was used and Wilcoxon signed-rank, Mann-Whitney *U*, or Kruskal-Wallis tests were used for non-normally distributed continuous variables. For within-patient comparison of variables over time, paired analysis was conducted. For the prediction of treatment response, response to PegIFN therapy and off-treatment response rates, logistic regression analyses were calculated. The level of significance was set at *p* <0.05. For graphical representation, the R package ‘ggplot2’ was used and *p* values were mapped as follows: ns, not significant; ∗*p* <0.05, ∗∗*p* <0.01, ∗∗∗*p* <0.001, and ∗∗∗∗*p* <0.0001.

### Ethics

The study was conducted in concordance with the principles of the Declaration of Helsinki and approved by the Ethics Committee of the Medical University of Vienna (ethics committee numbers: 1515/2020 & 2139/2021). Written informed consent was obtained from patients with CHD treated at the Vienna General Hospital, Klinik Ottakring, and the Medical University of Graz (recruiting 75.4% of included patients), whereas written informed consent was waived by the Institutional Review Board (IRB) for the retrospective inclusion of the remaining patients.

## Results

### Study cohort

In total, 61 patients living with CHD started BLV treatment at one of the participating centers between August 2019 and December 2024 and, thus, were included in the study. The median age was 45.0 (IQR 37.0–55.0) years, and the cohort was predominantly male (60.7%). Median BMI was 25.2 (IQR 21.9–29.3) kg/m^2^. Diabetes mellitus was present in 6.6% of patients. Daily alcohol consumption was reported by 6.6% of patients. At BL, ALT was 73.0 (IQR 45.0–123.0) U/L. The median LSM was 13.2 (IQR 9.1–18.8) kPa with 39 patients (69.6%) showing ≥10 kPa. Fifty-six patients (91.8%) received nucleos(t)id analog (NA) treatment. Almost half of patients (44.3%) had undergone previous treatment with PegIFN for CHD. Overall, 42 patients (68.9%) had already progressed to ACLD at the time of BLV initiation, and seven had decompensated disease. The overall BL characteristics, as well as the comparison between patients receiving BLV monotherapy and those receiving PegIFN add-on therapy, are presented in Table 1.

**Table 1:** Baseline characteristics.

Baseline characteristic	Overall (n = 61)	BLV monotherapy (n = 42)	PegIFN add-on (n = 19)	*p* value
Age, median yr (IQR)	45.0 (37.0–55.0)	49.0 (36.8–56.0)	42.0 (37.0–52.0)	0.308
Female sex, n (%)	24 (39.3)	19 (45.2)	5 (26.3)	0.161
BMI, median kg/m^2^ (IQR)	25.2 (21.9–29.3)	25.4 (22.2–30.1)	23.9 (20.8–28.6)	0.925
Diabetes, n (%)	4 (6.6)	3 (7.2)	1 (5.3)	0.793
Significant alcohol consumption, n (%)	4 (6.6)	3 (7.1)	1 (5.3)	0.784

**Liver disease severity**				
ACLD, n (%)	42 (68.9)	32 (76.2)	10 (52.6)	0.066
LSM, median kPa (IQR)∗	13.2 (9.10–18.8)	13.2 (9.6–20.0)	12.7 (7.0–16.4)	0.899
≥10 kPa, n (%)	39 (69.6)	30 (71.4)	9 (47.4)	0.070
≥15 kPa, n (%)	21 (37.5)	17 (40.5)	4 (21.1)	0.139
≥25 kPa, n (%)	7 (13.5)	5 (11.9)	2 (10.5)	0.876
CAP, median dB/m (IQR)†	221 (184–281)	225 (189–286)	200 (150–250)	0.707
S0, n (%)	22 (64.7)	14 (33.3)	8 (42.1)	
S1	2 (5.9	2 (4.8)	0 (0.0)	
S2	1 (2.9%)	1 (2.4	0 (0.0)	
S3	9 (26.5)	7 (16.7)	2 (10.5)	
Liver biopsy available, n (%)	29 (47.5)	22 (52.4)	7 (36.4)	0.832
F0-1	3 (10.3)	2 (4.8)	1 (5.3)	
F2	4 (13.8)	3 (7.1)	1 (5.3)	
F3	10 (34.5)	8 (19.0)	2 (10.5)	
F4	12 (41.4)	9 (21.4)	3 (15.8)	
MELD, median points (IQR)	8 (7–10)	8 (7–11)	8 (6–10)	0.480
ELF test, median points (IQR)‡	10.8 (9.7–11.8)	10.9 (9.9–12.2)	9.7 (9.3–11.2)	0.311
Decompensation, n (%)	7 (11.5)	4 (9.5)	3 (15.8)	0.477

**Virologic characteristics at baseline**				
HDV-RNA, median log_10_ copies/ml (IQR)	5.20 (3.92–5.72)	5.06 (3.68–5.57)	5.61 (5.04–6.12)	0.233
ALT, median IU/L (IQR)	73.0 (45.0–123.0)	70.5 (41.8–121.5)	91.0 (60.0–132.0)	0.172
HIV coinfection, n (%)	4 (6.6)	2 (4.8)	2 (10.5)	0.400
Anti-HCV antibodies, n (%)	5 (8.2)	3 (7.1)	2 (10.5)	0.656

**Hepatitis B characteristics**				
HBV-DNA >2,000 IU/ml, n (%)	3 (4.9)	1 (2.4)	2 (10.5)	0.171
HBsAg, median log_10_ IU/ml (IQR)	3.89 (3.40–4.16)	3.79 (3.21–4.24)	3.97 (3.46–4.15)	1.000
HbeAg positive, n (%)	6 (9.8)	5 (11.9)	1 (5.3)	0.578
NUC treatment, n (%)	56 (91.8)	38 (90.5)	18 (94.7)	0.574

**Hepatitis D treatment regimens**				
Previous PegIFN treatment, n (%)	27 (44.3)	22 (52.4)	5 (26.3)	0.058
BLV treatment, n (%)	61 (100.0)	42 (100.0)	19 (100.0)	1.000
BLV duration, median months (IQR)	29 (15.8–45.3)	27.1 (13.3–39.3)	37.0 (25.6–53.8)	0.233
PegIFN add-on to BLV, n (%)	19 (31.1)	0 (0.0)	19 (100.0)	—
PegIFN add-on at BLV treatment month, median (IQR)	10.5 (7.0–19.0)	0.0 (0.0–0.0)	10.5 (7.0–19.0)	—
PegIFN add-on duration, median months (IQR)	10.0 (8.0–12.0)	0.0 (0.0–0.0)	10.0 (8.0–12.0)	—

ACLD, advanced chronic liver disease; ALT, alanine aminotransferase; BLV, bulevirtide; CAP, controlled attenuation parameter; ELF, enhanced liver fibrosis; FIB-4, Fibrosis-4; HDV, hepatitis D virus; HDV, hepatitis D virus RNA; LSM, liver stiffness measurement; MELD, Model for End-Stage Liver Disease; NUC, nucleos(t)ide analog; PegIFN, pegylated interferon alfa-2a; VITRO, von Willebrand factor antigen to platelet ratio.

∗ LSM data available in 56 (91.8%) patients.

† CAP data available in 34 (55.7%) patients.

‡ ELF data available in 22 (36.1%) patients.

### Response rates to BLV

**Fig. 1:**
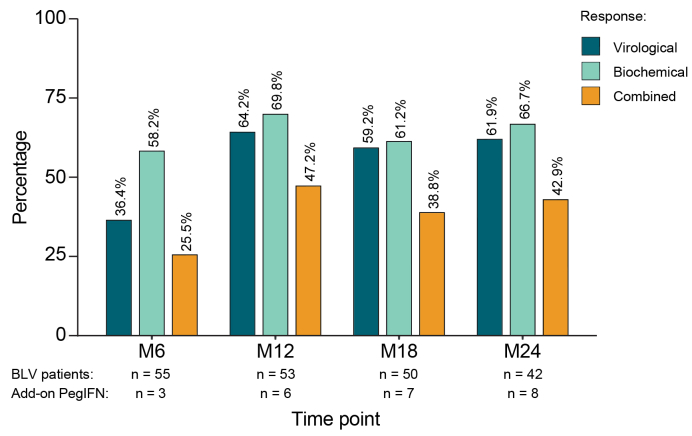
Treatment response to BLV with or without PegIFN add-on in the full cohort of 61 patients with CHD. BLV, bulevirtide; CHD, chronic hepatitis D; M, month; PegIFN, pegylated interferon alfa-2a.

The median observed duration of treatment with BLV was 29.0 (IQR 16.0–45.0) months. BR, VR, and CR to BLV treatment increased with treatment duration but appeared to plateau at M12 (Fig. 1). VR was achieved by 36.4% at M6, 64.2% at M12, and 61.9% at M24. BR was commonly achieved with 56.4% at M6, 69.8% at M12, and 66.7% at M24. CR was achieved by 25.5% of patients at M6, which increased to 47.2% by M12 and further plateaued at 42.9% at M24. For the dynamic responses during the first 24 months, see Fig. S1. Of the 61 patients, 42 received BLV monotherapy throughout the observation period, whereas 19 patients were treated with PegIFN add-on therapy at some point during their BLV course. Among the 42 patients with BLV monotherapy, VR at M6, M12, and M24 was 36.1%, 60.0%, and 56.0%, respectively. BR at M6, M12, and M24 was 63.9%, 77.1%, and 56.0%, resulting in CR rates of 30.6% at M6, 51.4% at M12, and 44.0%, respectively at M24. No difference in response rates was found between patients with and without previous PegIFN therapy before BL (Table S1).

HDV-RNA levels decreased significantly within the first 6 months of BLV treatment in the overall cohort (n = 61, median BL HDV-RNA 5.20 [IQR 3.92–5.72] log_10_ copies/ml *vs.* M6 HDV-RNA 3.18 [IQR 2.52–3.87 ] log_10_ copies/ml, *p* <0.001) and declined even further by M24 (median M24 HDV-RNA 2.41 [IQR 0.00–3.60] log_10_ copies/ml, *p* <0.001) (Fig. S2). ALT levels also decreased significantly at M6 (BL median ALT 73.0 [IQR 45.0–123.0] IU/L *vs.* M6 ALT 41.0 [IQR 29.0–62.0] IU/ml, *p* <0.001) and M24 (median M24 ALT 41.0 [IQR 25.0–51.5] IU/ml, *p* <0.001). No correlation could be found between LSM amelioration and ALT or HDV-RNA declines (Tables S2 and S3). Fig. S3 presents HDV-RNA and ALT kinetics stratified by the treatment regimen (BLV monotherapy *vs.* BLV+PegIFN add-on). Detailed information on biomarker dynamics during BLV treatment is given in the supplemental data online.

### Treatment discontinuation

During the treatment, a total of 20 patients (32.8%) achieved HDV-RNA TND. Among these, six patients had received add-on therapy with PegIFN. Consequently, the rate of HDV-RNA TND was 31.6% (six out of 19 patients) in the PegIFN add-on group and 33.3% (14 out of 42 patients) in the group treated with BLV monotherapy only. In 10 patients, BLV therapy was electively discontinued, three of whom had received add-on PegIFN treatment. The median time on BLV treatment before treatment discontinuation was 23.0 (IQR 12.0–29.0) months. The median on-treatment time of HDV-RNA TND among these 10 patients was 6.5 (IQR 5.3–13.5) months, whereas the seven patients receiving BLV monotherapy were HDV-RNA TND for a median of 6.0 (IQR 4.0–10.5) months.

Three patients (30.0%) relapsed after treatment discontinuation (Fig. S4). These patients had undetectable HDV-RNA for 3, 4, and 6 months before treatment withdrawal, respectively, and none received add-on PegIFN before discontinuation. All patients with virological relapse were reinitiated on BLV. In two patients, a second treatment discontinuation was attempted after HDV-RNA had remained TND for 18 and 19 months, respectively. Neither patient exhibited viral relapse during a follow-up period of 6 months after the second treatment discontinuation. One of these two patients received add-on PegIFN during the second course of BLV therapy. The remaining seven patients remained HDV-RNA TND for 36.0 (IQR 17.5–37.0) months without BLV.

Nine patients stopped BLV treatment without achieving HDV-RNA TND for other reasons, such as allergic reaction (n = 1, this patient was treated with hyposensibilization therapy and continued BLV treatment afterwards^22^), pruritus (n = 1, restarted), insurance issues (n = 2, both restarted, one later received an orthotopic liver transplantation), liver transplantation (n = 1, without relapse so far), wish for children (n = 1, acute ALT flare after discontinuation, restarted within 8 weeks), or initiation of other treatments for CHD within clinical trials (n = 3). The clinical courses of each patient in the observed timeframe are depicted in Fig. S5.

### Add-on PegIFN treatment

Nineteen patients (31.1%) received add-on PegIFN together with BLV. The median time of BLV monotherapy before PegIFN add-on was 10.5 (IQR 7.0–19.0) months and the median duration of add-on PegIFN treatment was 10.0 (IQR 8.0–12.0) months. Six patients (31.6%) began add-on therapy with the full PegIFN dose of 180 μg/week, whereas 13 patients (68.4%) started with a reduced dose, resulting in a median PegIFN dose of 135.0 (IQR 90.0–180.0) μg per week. In three of the six patients with the standard dosage, dose reduction during therapy was required because of dose-limiting side effects (mainly cytopenia and fatigue).

Overall, 14 patients (73.7%) discontinued the add-on therapy before the planned 48-week duration. The most common reasons for early discontinuation of PegIFN were adverse effects, including fatigue, leukopenia, and arthralgia, which were reported in nine patients. In addition, one patient discontinued PegIFN therapy because of logistical difficulties in drug delivery and, in another case, treatment was terminated after 7 months because of nonresponse. In three patients, the reason for PegIFN discontinuation was unknown. Five patients (26.3%) completed at least 48 weeks of PegIFN add-on therapy, of whom two received it for ≥96 weeks. No significant difference in VR was observed between patients with PegIFN treatment duration ≥48 weeks compared with those treated for <48 weeks at M12 (80.0% *vs.* 71.4%; *p* = 0.709) and M18 (100.0% *vs.* 61.5%; *p* = 0.103) of the overall BLV treatment course. At M24 of the overall treatment duration, VR rates were numerically higher in patients with ≥48 weeks of add-on treatment; however, this effect did not reach statistical significance (100.0% *vs.* 58.3%; *p* = 0.086). The median decline in HDV-RNA from initiation of PegIFN add-on therapy to the last available measurement did not differ significantly between the two groups (≥48 weeks: 3.04 [IQR 2.32–4.13] log_10_ copies/ml *vs.* <48 weeks: 0.99 [IQR 0.00–2.32] log_10_ copies/ml; *p* = 0.284). However, these comparisons lack statistical power.

**Fig. 2:**
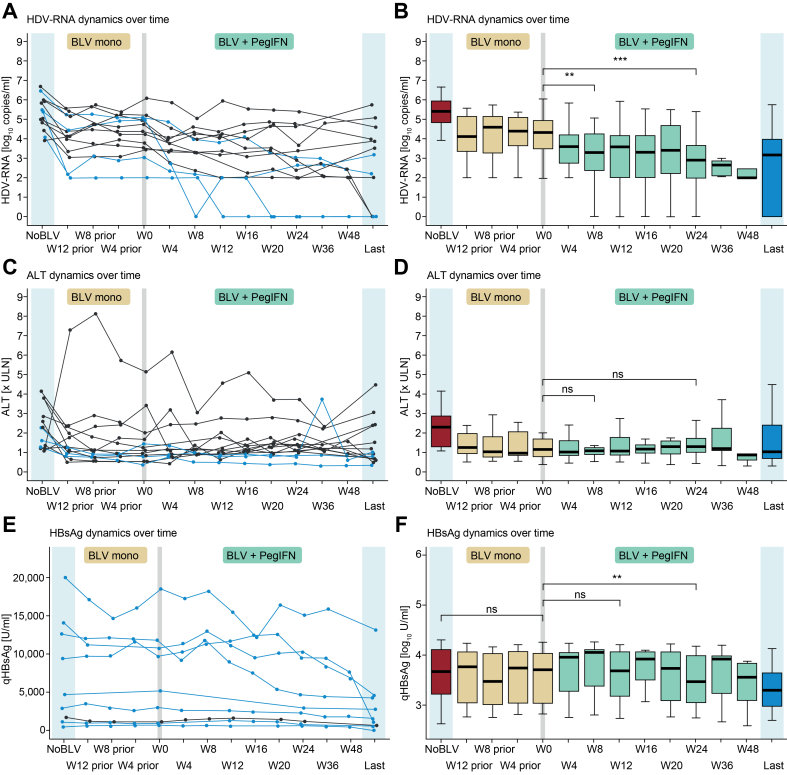
Effects of add-on PegIFN treatment on HDV-RNA, ALT, and HBsAg levels. Data of the parameters of interests are shown for 13/19 patients for whom these data were available. ‘W0’ marks the beginning of add-on PegIFN. BLV treatment-naive values are shown on the left (‘No BLV’), whereas the last available parameters after PegIFN discontinuation (except for one patient, who was treated with combination therapy until liver transplantation: last HDV-RNA = 2.0 log_10_ copies/ml, last ALT = 46 IU/ml, last HBsAg not available) are shown on the right (‘Last’). (A,B) HDV-RNA dynamics over time. Addition of PegIFN resulted in a decline in HDV-RNA levels ≥2 log in four patients (30.8%) by W24 compared with W0 (blue lines in A). (C,D) ALT levels over time. Three patients (23.1%) had ALT levels within the normal range by W24 (blue lines in C), which increased to seven (53.8%) at the last follow-up. (E,F) HBsAg levels over time. All but one patient who received add-on PegIFN showed a decline in HBsAg levels by W24 (blue lines in E). RNA acid; PegIFN, pegylated interferon alfa-2a; qHBsAg, quantitative HBsAg; W, week.

Thirteen patients who received add-on PegIFN treatment had detailed information regarding ALT and HDV-RNA available in 4-week intervals from 12 weeks before to 24 weeks after PegIFN initiation. These patients usually did not achieve VR or BR under BLV monotherapy (Fig. 2).^17^ Addition of PegIFN resulted in a significant further decline of HDV-RNA already by Week 8 (W0 HDV-RNA 4.36 [IQR 3.53–4.96] log_10_ copies/ml *vs.* W8 HDV-RNA 3.30 [IQR 2.36–4.23] log_10_ copies/ml, *p* = 0.003) and Week 24 (W24 HDV-RNA 2.89 [IQR 2.00–3.66] log_10_ copies/ml, *p* = 0.005). The median log_10_ decline by Week 24 of PegIFN add-on treatment compared with the last on-BLV monotherapy HDV-RNA value was 1.65 (IQR 0.81–2.11) copies/ml. In four patients, PegIFN add-on therapy resulted in an HDV-RNA decline of ≥2 log_10_ at Week 24, and two patients achieved HDV-RNA TND after 24 weeks of treatment. The median duration of add-on PegIFN therapy in these patients was 9.5 (IQR 8.0–14.3) months, and the observed effect on HDV-RNA suppression was maintained even after PegIFN discontinuation. Overall, the last available HDV-RNA, assessed after a median of 13.5 (IQR 10.0–24.5) months following PegIFN discontinuation, remained significantly lower than baseline levels before initiation of combination therapy (last HDV-RNA 3.17 [IQR 0.00–3.97] log_10_ copies/ml, *p* = 0.027 compared with W0). For all patients, the last available HDV-RNA measurement was obtained after PegIFN withdrawal, except for one patient, who continued BLV + PegIFN until liver transplantation. Upon PegIFN initiation, an increase in ALT levels was observed in some patients, but overall, PegIFN addition did not significantly affect ALT levels (W0 ALT 48.0 [IQR 28.0–84.0] IU/L *vs.* W24 55.0 [IQR 36.0–85.0] IU/L, *p* = 0.972).

Levels of HBsAg were not affected by BLV monotherapy (Fig. 2). Addition of PegIFN resulted in a significant reduction in HBsAg levels by Week 24 of combined treatment (W24 HBsAg 3.46 [IQR 3.05–3.98] log_10_ IU/ml *vs.* W0 HBsAg 3.71 [IQR 3.04–4.03] log_10_ IU/ml, *p* = 0.008), corresponding to a median reduction after 24 weeks of combined treatment of 0.08 (IQR 0.02–0.12) log_10_ IU/ml. None of the patients achieved an HBsAg <100 IU/ml at week 24. The observed decline in HBsAg was maintained and even more pronounced at the latest follow-up (FU) after PegIFN discontinuation (‘last’ median HBsAg 3.18 [IQR 2.78–3.63] log_10_ IU/ml, *p* = 0.004 *vs.* W0).

### Predictors of response rates to PegIFN therapy and off-treatment response

In logistic regression analyses, the predictive value of BL characteristics and markers of hepatic dysfunction for achieving VR at Week 24 of PegIFN add-on therapy, as well as for off-treatment response, was evaluated. In terms of VR to PegIFN add-on therapy, none of the assessed BL variables (including age, BMI, presence of ACLD, MELD, LSM, HDV-RNA or ALT levels) or treatment-related parameters, such as the duration of preceding BLV monotherapy before starting the add-on treatment, PegIFN dose, or duration of PegIFN add-on therapy, were identified as significant predictors (Table S4). Similarly, no baseline or treatment-related factors were associated with off-treatment response (Table S5). Predictors for VR, BR, and CR at M6 and M12 of BLV therapy are described in the supplemental data online.

### Follow-up and clinical outcomes

Two patients developed HCC while taking BLV and did not achieved VR under antiviral therapy. Furthermore, two patients underwent orthotopic liver transplantation during BLV therapy, one because of decompensated ACLD and one because of HCC. BLV was discontinued after transplantation, and no relapse was observed under HBV-targeted prophylaxis. Two patients with advanced decompensated cirrhosis in whom BLV was started as a bridge-to-transplant treatment died shortly after treatment initiation before the evaluation for transplant could be finished: One patient died of cardiac arrest and one died of bleeding-associated acute-on-chronic liver failure after 2 and 5 weeks of BLV, respectively. Both events were considered unrelated to antiviral treatment.

One patient with advanced compensated cirrhosis and clinically significant portal hypertension (CSPH) had been on long-term dialysis owing to end-stage kidney disease before BLV initiation. BLV was applied three times per week following dialysis, initially. No adverse events occurred, and bile acid levels were within the normal range. Given the lack of VR to treatment at M12, the dosage was increased to the standard dose of 2 mg per day. Thereupon, the patient achieved BR and showed a 1.39 log_10_ decrease in HDV-RNA compared with BL at M24. The patient underwent successful kidney transplantation after 26 months of treatment and continued BLV thereafter.

## Discussion

In this report summarizing long-term treatment outcomes observed within the Austrian CHD cohort exposed to BLV ± PegIFN treatment, we observed increasing response rates to BLV treatment over time. BLV has been licensed based on preliminary results of phase II studies. Following its approval, the primary endpoint results observed within the MYR 301 study have been published, where similar response rates to our real-world cohort were observed at M12 of treatment.^21^ Other real-world cohort studies have shown similarly encouraging results.^17^^,^^23^^,^^24^ In a recent collaborative European study, satisfying response rates were demonstrated in a multicenter cohort of 244 patients who had already developed cirrhosis before BLV treatment initiation.^23^ Notably, CR rates to BLV were consistently lower than both VR and BR rates, indicating that HDV-RNA suppression and ALT normalization do not universally occur synchronously. This observation aligns with findings from both clinical trials^25^ and real-world cohorts^23^ and likely reflects distinct underlying mechanisms, given that ALT normalization can result from reduced intrahepatic inflammation, whereas HDV-RNA decline is directly related to inhibition of viral entry and indirect effects on replication.^26^ However, despite the apparent link between longer treatment duration and increasing response rates in clinical trials and real-world cohorts, some patients fail to achieve relevant reductions in HDV-RNA or even ALT. In our cohort, we observed a plateau in response, as evident from >30% and >50% of patients who failed to achieve VR and CR, respectively. In part, this could be explained by selection bias because difficult-to-treat patients with advanced cirrhosis had been prioritized for BLV treatment in Austria after the novel antiviral therapy became available, thereby resulting in an over-representation of these vulnerable patients at later timepoints investigated in our study. Regardless of the causes of a suboptimal response in a considerable number of patients undergoing long-term BLV therapy, we generally pursue HDV-RNA suppression as the most desirable endpoint of therapy, because persisting HDV viremia appears to be the strongest predictor for adverse outcomes and disease progression in CHD besides the stage of chronic liver disease.^27^

Thus, most Austrian hepatitis clinics have adopted a response-guided approach of adding PegIFN to BLV in patients with nonresponse to BLV monotherapy, as reported in a previous study conducted in fewer patients recruited at a smaller number of centers.^17^ In the current report, we substantiate the concept of response-guided therapy in CHD by providing granular data demonstrating that add-on PegIFN can cause a further decline in HDV-RNA viral load of almost 2 log_10_ levels in suboptimal responders to BLV or patients who reach a virological plateau. Notably, this strategy was also applied in patients with a plateauing virological response rather than being limited to cases of primary maintained nonresponse. From a clinical perspective, a plateau can identify patients in whom BLV exerts partial antiviral activity but fails to achieve further viral decline, thereby representing a window of opportunity for combination therapy. Escalation of treatment at this stage might be preferable to prolonged monotherapy without further HDV-RNA declines or even relapse. Importantly, our current data demonstrate that those advantageous further declines persist even after PegIFN is discontinued. Furthermore, we observed no relevant/sustained effects on ALT, whereas slight decreases in HBsAg levels were observed in patients undergoing combined therapy. The observed synergistic effect of BLV and PegIFN is explained by an elegant experimental study demonstrating that, although BLV blocks the NTCP-based spread of HDV from hepatocyte to hepatocyte, interferon additionally blocks the cell division-based transfer of HDV-RNA.^28^^,^^29^ In line with this, the landmark MYR 204 study demonstrated that a considerable number of patients achieved HDV-RNA TND at the end of combined treatment, which was maintained by one out of three patients treated with BLV (2 mg) and PegIFN.^16^ Meanwhile, HDV-RNA relapses were frequently observed in long-term FU studies among the patients recruited in the HIDIT-II trial assessing the effect of PegIFN monotherapy.^30^^,^^31^ Limited data exist on the sustainability of HDV-RNA TND achieved under BLV monotreatment, although sharp and immediate rebounds in HDV-RNA were observed following BLV discontinuation in (viremic) patients included in the MYR 202 study and our group has reported relapses even despite long-term HDV-RNA TND.^32^^,^^33^

Notably, HDV-RNA quantification in this study was performed by two different assays: the in-house assay of the Medical University of Vienna was used for 55 patients, and the RoboGene HDV Quantification Kit 2.0 was used for six patients. Although this approach is appropriate for assessing declines of ≥2 log_10_, it might introduce variability when evaluating HDV-RNA TND, because the sensitivity of the two assays differ in their limits of quantification (LOQ) and detection (LOD). Thus, a certain level of uncertainty in interpreting TND rates must be acknowledged as a limitation of this study. Nevertheless, the application of a previously established conversion factor^17^ enabled comparability of results, given that the LOD did not differ substantially between the two assays used (8 IU/ml *vs.* 3–4 IU/ml).

In line with the preliminary W240 results of the MYR301 study,^34^ in which 36% of patients maintained undetectable HDV-RNA at FU Week 96 after treatment cessation, our data confirm that a subset of patients can achieve sustained HDV-RNA TND upon BLV (±PegIFN) treatment. In our cohort, 10 patients discontinued BLV after a median of 23 months, and 70% of these maintained HDV-RNA TND for a median of 36 months, a proportion that appears to be higher than the rate of sustained undetectable HDV-RNA reported in MYR301, although treatment durations in our cohort were not standardized.^34^ Similar to MYR301,^36^ all three relapses occurred within the first year after treatment discontinuation, emphasizing that relapse is more likely to be early after treatment cessation. Importantly, all three patients were only suppressed for less than 6 months. BLV was reinitiated in those three patients, and all achieved HDV-RNA TND again. Two even discontinued treatment again and remain HDV-RNA TND to date. Of note, none of the three had previously undergone combined BLV and PegIFN treatment, whereas three out of four patients who did not relapse following BLV discontinuation received PegIFN add-on treatment. Although our data provide interesting insights into BLV discontinuation, we acknowledge that no consistent stopping rules or reliable predictors of response could be identified. Of note, our definition of sustained VR (*i.e.*, ≥24 weeks) used to guide BLV discontinuation was relatively short when considering the potentially beneficial impact of a longer duration of HDV-RNA suppression (*i.e.*, ≥96 weeks) before treatment cessation.^34^ However, these data were not available when this study was planned. In light of the MYR 301 study results,^34^ decisions regarding BLV cessation should be individualized and might be limited to selected patients with long-term HDV-RNA TND, and in whom continued close monitoring with the option for BLV reinitiation is feasible. Future studies are needed to define safe stopping criteria for BLV. Furthermore, given the high cost and requirement for s.c. administration, the practical and economic aspects of indefinite BLV therapy warrant further evaluation.

Two out of three patients among our cohort had already developed ACLD. Levels of biomarkers of systemic inflammation increase as chronic liver disease progresses, which, in turn, might also be driver of further deterioration.[35], [36], [37], [38] Etiological treatment reduces systemic inflammation in viral hepatitis^39^^,^^40^ as well as alcohol-related liver disease.^41^ In our cohort, we observed not only a significant reduction in biomarkers reflecting hepatic (ALT) but also a systemic inflammation (C-reactive protein [CRP] and procalcitonin [PCT]) upon treatment, which steadily declined over time. Furthermore, reductions in non-invasive markers of liver fibrosis, such as LSM and ELF score, were observed under therapy. Taken together with the low number of adverse hepatic events observed during long-term therapy, BLV treatment appears prone to beneficially impacting the natural history of CHD, but prospective studies confirming its prognostic benefits are needed. In terms of safety, although bile acids increased with BLV treatment initiation, levels stabilized and only one patient (1.6%) temporarily paused treatment because of pruritus. A previously published anaphylactic reaction to BLV was the only serious adverse event related to BLV observed within our cohort, which, according to the literature, appears to be a rare complication of treatment.^22^

Despite the discussed limitations that are inherent to registry-based real-world cohort studies, our study has several strengths. First, we provide insights from a nationwide registry that covers almost all Austrian patients exposed to BLV therapy since it became available in our country. Thus, even though the cohort size is limited, and treatment regimens and durations differ, we provide unique insights into treatment outcomes observed within a contemporary cohort of patients with CHD representing all stages of chronic liver disease, including patients with decompensated cirrhosis and many difficult-to-treat patients who had been exposed to previous PegIFN treatment. Moreover, we provide evidence corroborating the long-term benefit of add-on therapy with PegIFN even after discontinuation of PegIFN and we report a considerable number of patients who maintained HDV-RNA TND after BLV ± PegIFN withdrawal, informing future clinical trial design. Lastly, despite intensive efforts aiming at suppression of HDV-RNA to undetectable levels, two out of three patients could not achieve this desirable endpoint in our study, highlighting the need for innovative treatments being explored in clinical studies and compassionate use programs.^42^

In summary, in our Austrian cohort of patients with CHD treated with BLV, we report high response rates after a median of 2 years of treatment alongside excellent tolerability of BLV, even in patients with advanced compensated and even decompensated chronic liver disease. In suboptimal BLV responders, add-on PegIFN was associated with a significant and seemingly sustainable decline in HDV-RNA and HBsAg, with effects that were sustained after treatment discontinuation in a subset of patients, indicating a meaningful contribution to viral infection control. Selected patients achieving long-term (>6-12 months) HDV-RNA suppression could qualify for finite BLV ± PegIFN treatment, and virological relapse (observed in three out of 10 patients with TND) responded well to BLV re-exposure. Finally, an amelioration of biomarkers reflecting fibrosis and systemic inflammation alongside the low number of liver-related complications under BLV ± PegIFN therapy substantiates the hypothesis that HDV-RNA suppression indicates clinical benefit in patients with CHD with ACLD.

## Abbreviations

ACLD, advanced chronic liver disease; ALT, alanine aminotransferase; APRI, aspartate aminotransferase (AST) to platelet ratio index; AST, aspartate aminotransferase; BL, baseline; BLV, bulevirtide; BR, biochemical response; CAP, controlled attenuation parameter; CR, combined response; CRP, C-reactive protein; CHB, chronic HBV; CHD, chronic hepatitis D; CSPH, clinically significant portal hypertension; ELF, enhanced liver fibrosis; FIB-4, Fibrosis-4; FU, follow-up; HCC, hepatocellular carcinoma; HDV, hepatitis D virus; LOQ, limit of quantification; LOD, limit of detection; LSM, liver stiffness measurement; M6/M12/M18/M24, month 6, 12, 18, 24; MELD, model for end-stage liver disease; NA, nucleos(t)id analog; NTCP, sodium taurocholate co-transporting polypeptide; PCT, procalcitonin; PegIFN, pegylated interferon alfa-2a; TND, target not detectable; VR, virological response; W0, W24, etc., Week 0, Week 24, etc.

## Authors’ contributions

Research design: MS, MH, TR, MJ. Data acquisition: MS, MH, CS, MP, NP, NL, LH, LD, HL, IG, AMa, AMo, EA, VS, CM, SWA, MJ. Analysis of data: MS, MH, TR, MJ. Interpretation of data: all authors. Drafted the manuscript: MS, MH, TR, MJ.Critically revised: all other authors.

## Data availability statement

The data are available upon reasonable request to the corresponding author.

## Financial support

No specific funding was received for this study.

## Conflicts of interest

MS received travel support from MSD, Sandoz, BMS, AbbVie and Gilead; and speaking honoraria from BMS and Gilead; consulting fees from Gilead. MH received travel support from Gilead and Roche; and speaking honoraria from Gilead. CS received travel support from Gilead, Abbvie, Galápagos, and Gebro; speaking honoraria from Abbvie and Gilead; and payments for consulting from Gilead. MP served as a speaker and/or consultant and/or advisory board member for MSD, AbbVie, Intercept, and Gilead, and received travel support from Gilead and AbbVie. NP received travel support from Gilead. LH received travel support from AbbVie. AMa received grant support from Abbvie and Gilead; speaking honoraria from Abbvie, Gilead, Janssen, Roche, Intercept, and MSD; consulting/advisory board fees from Abbvie, Gilead, Janssen, Roche, Intercept, Norgine, and MSD; and travel support from Abbvie, Gilead and Roche. AMo received research support from AbbVie and Takeda under the framework of the Christian Doppler Research Society; received further consultation fees and/or speaker honoraria from AbbVie, Merck Sharp & Dohme, Takeda, Janssen-Cilag, Amgen, Sandoz, Nestlé, Ferring, Falk, and Pfizer. EA received travel support and advisory fees from Gilead. VS received grant support from Gilead, Immundiagnostik, Merz Therapeutics, Lactosan, Winclove, Institut Allergosan and received speakers honoraria/travel support and consulting/advisory board honoraria from Merz Therapeutics, Institut Allergosan, Alnylam, Sanofi, Gilead, Tillotts, Böhringer Ingelheim. MT received grant support from Albireo, Alnylam, Cymabay, Falk, Gilead, Intercept, MSD, Takeda and UltraGenyx; honoraria for consulting from AbbVie, Albireo, Boehringer Ingelheim, BiomX, Falk, Genfit, Gilead, Hightide, Intercept, Janssen, MSD, Novartis, Phenex, Pliant, Regulus, Shire, and Siemens; speaker fees from Albireo, Bristol Myers Squibb, Falk, Gilead, Intercept, Madrigal, and MSD as well as travel support from AbbVie, Falk, Gilead, and Intercept. He is also co-inventor on patents on the medical use of norUDCA/norucholic acid filed by the Medical University of Vienna. MM served as a speaker and/or consultant and/or advisory board member for AbbVie, Collective Acumen, Echosens, Gilead, Takeda, and W.L. Gore & Associates and received travel support from AbbVie and Gilead. IG served as a speaker and/or consultant and/or advisory board member for AbbVie, Gilead, Ipsen, Galapagos, Astra Pharma, Intercept, Falk and received travel support from Roche, Gilead and AbbVie. HZ received speaker honoraria from the Abbvie, Bayer, BMS, Falk Foundation, Gilead, Intercept, Merck, MSD, Novartis, Pierre-Fabre, Pharmacosmos, and Vifor; he has advised for Abbvie, Bayer, Eisai, Gilead, Intercept, MSD, Novartis, Novo Nordisk, Shire, Pierre-Fabre, Pharmacosmos, and Vifor. He further received travel grants from Abbvie, Bayer, Gilead, and Intercept, and research grants from Abbvie, Gilead, MSD, Novartis, Pharmacosmos, and Vifor. MG received grant support from Abbvie, Gilead, and MSD; speaking honoraria from Abbvie, Gilead, Janssen, Roche, Intercept, and MSD; consulting/advisory board fees from Abbvie, Gilead, Janssen, Roche, Intercept, Norgine, AstraZeneca, Falk, Shionogi, and MSD; and travel support from Abbvie and Gilead. TR served as a speaker and/or consultant and/or advisory board member speaking honoraria from AbbVie, Bayer, Boehringer-Ingelheim, Gilead, Intercept, MSD, Roche, Siemens, and W.L. Gore & Associates and received travel support from AbbVie, Boehringer-Ingelheim, Gilead, and Roche as well as grants/research support from AbbVie, Boehringer-Ingelheim, Gilead, Intercept, MSD, Myr Pharmaceuticals, Philips Healthcare, Pliant, Siemens, and W.L. Gore & Associates. MJ served as a speaker and/or consultant for Gilead, and has received unrestricted research grants from Gilead Sciences Inc. NL, LD, HL, AFT, CM and SWA have nothing to disclose.

Please refer to the accompanying ICMJE disclosure forms for further details.
