# Supplementary material for: Response-guided bulevirtide ± pegylated interferon alfa-2a: Long-term outcomes observed in the nationwide Austrian hepatitis D cohort study
Source: JHEP Rep. 2026 Mar 26;8(6):101835. doi: 10.1016/j.jhepr.2026.101835 (PMC13199667; doi:10.1016/j.jhepr.2026.101835)
Supplement: Multimedia component 1 [file mmc1.pdf]

Response-guided bulevirtide ± pegylated interferon alfa-2a treatment: long-term outcomes observed in the nationwide Austrian hepatitis D cohort study

Michael, Schwarz, Marlene, Hintersteininger, Caroline, Schwarz, Marlene, Panzer, Nikolaus, Pfisterer, Nina, Loschko, Lukas, Hartl, Livia, Dorn, Hermann, Laferl, Michael, Trauner, Albert, F. Stättermayer, Mattias, Mandorfer, Ivo, Graziadei, Andreas, Maieron, Alexander, Moschen, Elmar, Aigner, Vanessa, Stadlbauer, Christian, Madl, Stephan, W. Aberle, Heinz, Zoller, Michael, Gschwantler, Thomas, Reiberger, Mathias, Jachs

Table of contents

RESULTS

Systemic inflammation, bile acids and biomarkers of liver disease	Page 2

Predictors of treatment response to BLV	Page 3

SUPPLEMENTARY TABLES

Table S1	Page 4

Table S2	Page 5

Table S3	Page 5

Table S4	Page 6

Table S5.	Page 8

SUPPLEMENTARY FIGURES

Fig. S1	Page 8

Fig. S2	Page 9

Fig. S3	Page 10

Fig. S4	Page 11

Fig. S5	Page 12

RESULTS

Systemic inflammation, bile acids and biomarkers of liver disease

Paired values of biomarkers of systemic inflammation, i.e., C-reactive protein (CRP, available in n=54 including n=14 with add-on PEG-IFN), procalcitonin (PCT, available in n=20 including n=4 with add-on PEG-IFN), and interleukin 6 (IL6, available in n=20 including n=4 with add-on PEG-IFN) were available in a subset of patients. As shown in Supplementary Fig. S2, BLV ± PEG-IFN treatment induced a significant decrease in CRP at M6 (0.25 [IQR 0.1-0.29] mg/dL vs. BL: 0.29 [IQR 0.12-0.51] mg/dL, p=0.003) and even further at M24 (0.16 [IQR 0.06-0.29] mg/dL, p<0.001 vs. BL). Similarly, PCT decreased at M6 (0.05 [IQR 0.03-0.09] ng/mL vs. BL: 0.11 [IQR 0.08-0.16] ng/mL, p=0.003) and at M24 (0.03 [IQR 0.02-0.06] ng/mL, p=0.009 vs. BL). There was also a (non-significant) trend towards decreasing levels of IL6 (BL: 3.84 [IQR 2.72-4.67] pg/mL vs. M6: 3.18 [IQR 1.88-4.25] pg/mL, p=0.426; M24: 3.01 [IQR 1.80-4.03] pg/mL, p=0.301 vs. BL).

Bile acids increased significantly after BLV treatment initiation (paired results in n=17; M6: 20.0 [IQR 10.3-45.8] µmol/L vs. BL: 8.0 [IQR 4.3-14.4] µmol/l, p<0.001) but did not increase further during prolonged BLV treatment. Pruritus requiring temporary treatment withdrawal occurred in one patient, in whom BLV could be reintroduced upon supportive treatment and was thereupon well tolerated.

Liver stiffness significantly decreased under BLV treatment (BL: 13.2 [IQR 9.1-18.8] kPa, M6: 10.7 [IQR 7.4-14.7] kPa, p=0.003, M24: 8.7 [IQR 6.8-15.6] kPa, p<0.001 vs. BL). Similarly, ELF test (non-significantly) decreased (BL: 10.8 [IQR 9.7-11.8], M6: 9.8 [IQR 9.4-11.0], M24: 9.6 [IQR 9.2-10.4]; p=0.135 vs. BL). Furthermore, BLV treatment led to a significant decrease of quantitative IgG (BL: 1920 [IQR 1685-2198] mg/dL, M6: 1800 [1530-1880] mg/dL, p=0.042; M12: 1670 [IQR 1408-1842] mg/dL, p=0.004; M18 IgG 1630 [1230-1862] mg/dL, p=0.048; M24 1515 [1212-1820] mg/dL, p=0.193; all vs. BL).

Predictors of treatment response to BLV

In logistic regression analysis, the predictive utility of BL parameters for VR, BR, and CR at M6 and M12 was investigated. Higher ALT (coefficient [CE] -0.009 [standard error, SE, 0.004], z value -2.026, p=0.043), gGT (CE -0.015 [SE 0.007], z value -2.227, p=0.026), and HBsAg (CE -0.000 [SE 0.000], z value -2.242, p=0.025) levels associated with lower probability of achieving BR at M6. Higher levels of gGT (CE -0.023 [SE 0.008], z value -2.918, p=0.004) also associated with lower probability of achieving BR at M12, while BL ALT and HBsAg did not seem to impair the probability of achieving BR at later treatment timepoints. Other than that, no predictors of treatment response to BLV were identified. No predictors for achieving VR or CR were identified.

SUPPLEMENTARY TABLES

Table S1. Comparison of virological, biochemical, and combined response rates between patients with and without prior PEG-IFN therapy before initiation of BLV.

	Previous PEG-IFN therapy (n=27)	No previous PEG-IFN therapy (n=34)	p-value
**VR month 6, n (%)**	6 (22.2%)	14 (41.2%)	0.123
**BR month 6, n (%)**	14 (51.9%)	18 (52.9%)	0.984
**CR month 6, n (%)**	5 (18.5%)	9 (26.5%)	0.489
**VR month 12, n (%)**	12 (44.4%)	23 (67.6%)	0.094
**BR month 12, n (%)**	18 (667%)	20 (58.8%)	0.274
**CR month 12, n (%)**	11 (40.7%)	15 (44.1%)	0.967
**VR month 18, n (%)**	10 (37.0%)	19 (55.9%)	0.154
**BR month 18, n (%)**	15 (55.6%)	15 (44.1%)	0.204
**CR month 18, n (%)**	8 (29.6%)	11 (32.4%)	0.933
**VR month 24, n (%)**	10 (37.0%)	16 (47.1%)	0.735
**BR month 24, n (%)**	13 (48.1%)	15 (44.1%)	0.266
**CR month 24, n (%)**	8 (29.6%)	10 (29.4%)	0.650

Abbreviations: BR, biochemical response; CR, combined response; PEG-IFN, pegylated interferon alfa-2a; VR, virological response.

Table S2. Correlations of on-treatment liver stiffness measurement ameliorations with HDV-RNA declines.

Abbreviations: BL, baseline; Δ, delta; HDV, hepatitis D virus; LSM, liver stiffness measurement; M, month; RNA, ribonucleic acid.

	Δ HDV-RNA BL - M6, $\log_{10}$ copies/mL		Δ HDV-RNA BL - M12, $\log_{10}$ copies/mL		Δ HDV-RNA BL - M18, $\log_{10}$ copies/mL		Δ HDV-RNA BL - M24, $\log_{10}$ copies/mL	
	rho	p-value	rho	p-value	rho	p-value	rho	p-value
**Δ LSM BL - M6, kPa**	-0.95	0.594	-0.07	0.730	0.02	0.921	-0.13	0.561
**Δ LSM BL - M12, kPa**	-0.07	0.703	0.07	0.710	0.17	0.391	0.00	0.987
**Δ LSM BL - M18, kPa**	-0.07	0.687	0.04	0.839	0.16	0.455	0.04	0.874
**Δ LSM BL - M24, kPa**	-0.16	0.432	0.036	0.878	0.21	0.358	0.09	0.707

Table S3. Correlations of on-treatment liver stiffness measurement ameliorations with ALT declines.

Abbreviations: ALT, alanine aminotransferase; BL, baseline; Δ, delta; LSM, liver stiffness measurement; M, month.

	Δ ALT BL - M6, IU/L		Δ ALT BL - M12, IU/L		Δ ALT BL - M18, IU/L		Δ ALT BL - M24, IU/L	
	rho	p-value	rho	p-value	rho	p-value	rho	p-value
**Δ LSM BL - M6, kPa**	-0.03	0.845	-0.11	0.505	-0.14	0.425	-0.17	0.370
**Δ LSM BL - M12, kPa**	0.25	0.118	0.19	0.241	0.07	0.675	0.20	0.273
**Δ LSM BL - M18, kPa**	-0.20	0.252	-0.20	0.260	-0.15	0.407	-0.21	0.291
**Δ LSM BL - M24, kPa**	0.11	0.541	0.13	0.472	0.11	0.548	0.09	0.628

Table S4. Impact of baseline characteristics, parameters of hepatic dysfunction and PEG-IFN therapy on virologic response rates at week 24 of PEG-IFN add-on therapy. Univariate binary logistic regression models are shown. Since none of the parameters were significantly associated with response, no multivariate analysis was performed.

**Parameter of interest**	**Univariate (unadjusted) analysis**		
**Virologic response at W24 of PEG-IFN therapy**	**Exp(B)**	**95%CI**	**p-value**
**Age, years**	1.07	0.95-1.21	0.247
**BMI, kg/m^2**	1.05	0.77-1.43	0.767
**ACLD**	1.50	0.11-21.3	0.765
**PEG-IFN add-on BLV month**	1.10	0.93-1.31	0.263
**PEG-IFN dose, µg/week**	1.02	0.99-1.05	0.255
**PEG-IFN duration, months**	0.99	0.86-1.16	0.939
**BL HDV-RNA, log_{10} copies/mL**	1.00	1.00-1.00	0.517
**BL LSM, kPa**	1.15	0.96-1.37	0.137
**MELD, points**	1.12	0.58-2.20	0.730
**BL VITRO score, points**	2.54	0.62-10.36	0.194
**BL FIB-4, points**	1.03	0.44-2.37	0.953
**BL HBs Ag, log_{10} IU/mL**	1.00	1.00-1.00	0.339
**BL ALT, IU/L**	1.01	0.98-1.03	0.648
**W0 ALT, IU/L**	0.98	0.94-1.02	0.349
**W0 HDV-RNA, log_{10} copies/mL**	1.00	1.00-1.00	0.607

Abbreviations: ACLD, advanced chronic liver disease; ALT, alanine aminotransferase; BL, baseline; BLV, bulevirtide; BMI, body mass index; FIB-4, fibrosis-4 index; HBs Ag, hepatitis B s-antigen; HDV, hepatitis D virus; LSM, liver stiffness measurement; MELD, model for endstage liver disease; PEG-IFN, pegylated interferon alfa-2a; RNA, ribonucleic acid; VITRO, von Willebrand factor antigen to platelet ratio; W, week.

Table S5. Impact of baseline characteristics, parameters of hepatic dysfunction and PEG-IFN therapy on off-treatment response.

Parameter of interest	Univariate (unadjusted) analysis		
**Off-treatment response**	**Exp(B)**	**95%CI**	**p-value**
**Age, years**	1.05	0.94-1.17	0.401
**BMI, kg/m^2**	0.94	0.69-1.28	0.698
**ACLD**	3.00	0.12-73.64	0.501
**Previous PEG-IFN therapy**	0.67	0.04-11.29	0.779
**PEG-IFN add-on**	0.67	0.04-11.29	0.779
**BL HDV-RNA, $\log_{10}$ copies/mL**	1.00	1.00-1.00	0.573
**BL LSM, kPa**	1.09	0.94-1.26	0.258
**MELD, points**	0.94	0.42-2.08	0.873
**BL VITRO score, points**	0.76	0.27-2.32	0.674
**BL FIB-4, points**	1.78	0.40-7.84	0.447
**BL HBs Ag, $\log_{10}$ IU/mL**	1.00	1.00-1.00	0.583
**BL ALT, IU/L**	1.00	0.99-1.00	0.942
**HDV-RNA TND, months**	0.64	0.30-1.37	0.248
**BLV treatment duration, months**	0.97	0.86-1.10	0.672

Univariate binary logistic regression models are shown. Since none of the parameters were significantly associated with response, no multivariate analysis was performed.

Abbreviations: ACLD, advanced chronic liver disease; ALT, alanine aminotransferase; BL, baseline; BLV, bulevirtide; BMI, body mass index; FIB-4, fibrosis-4 index; HBs Ag, hepatitis B s-antigen; HDV, hepatitis D virus; LSM, liver stiffness measurement; MELD, model for endstage liver disease; PEG-IFN, pegylated interferon alfa-2a; RNA, ribonucleic acid; TND, target not detected; VITRO, von Willebrand factor antigen to platelet ratio; W, week.

SUPPLEMENTARY FIGURES

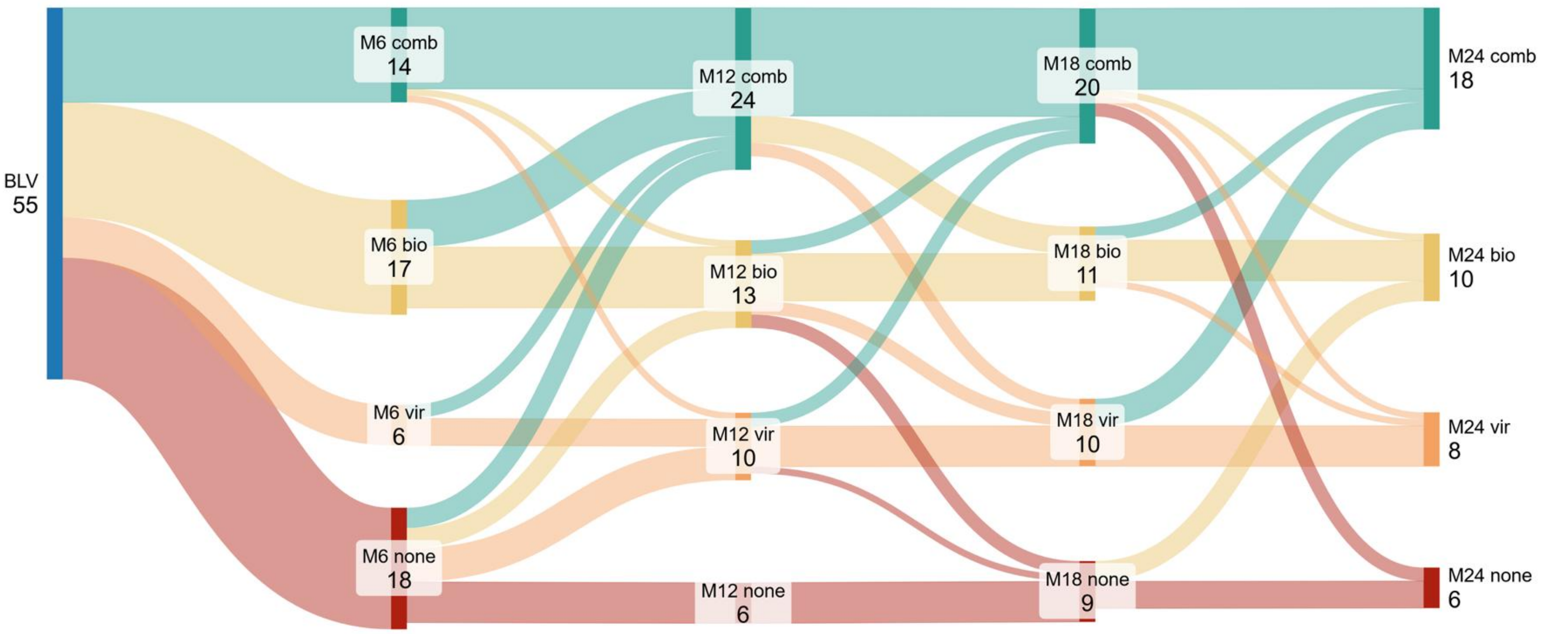

Fig. S1. Treatment response to BLV after 6, 12, 18 and 24 months (M6-24). Patients were assessed for virological, biochemical, and combined response at 6-month intervals. Patients who achieved virological response, usually also achieved biochemical response. Achieved responses were not permanent in all patients and some patients did not respond even after 24 months of treatment.

Abbreviations: bio, biological response; BLV, bulevirtide; comb, combined response; M, month, vir, virological response.

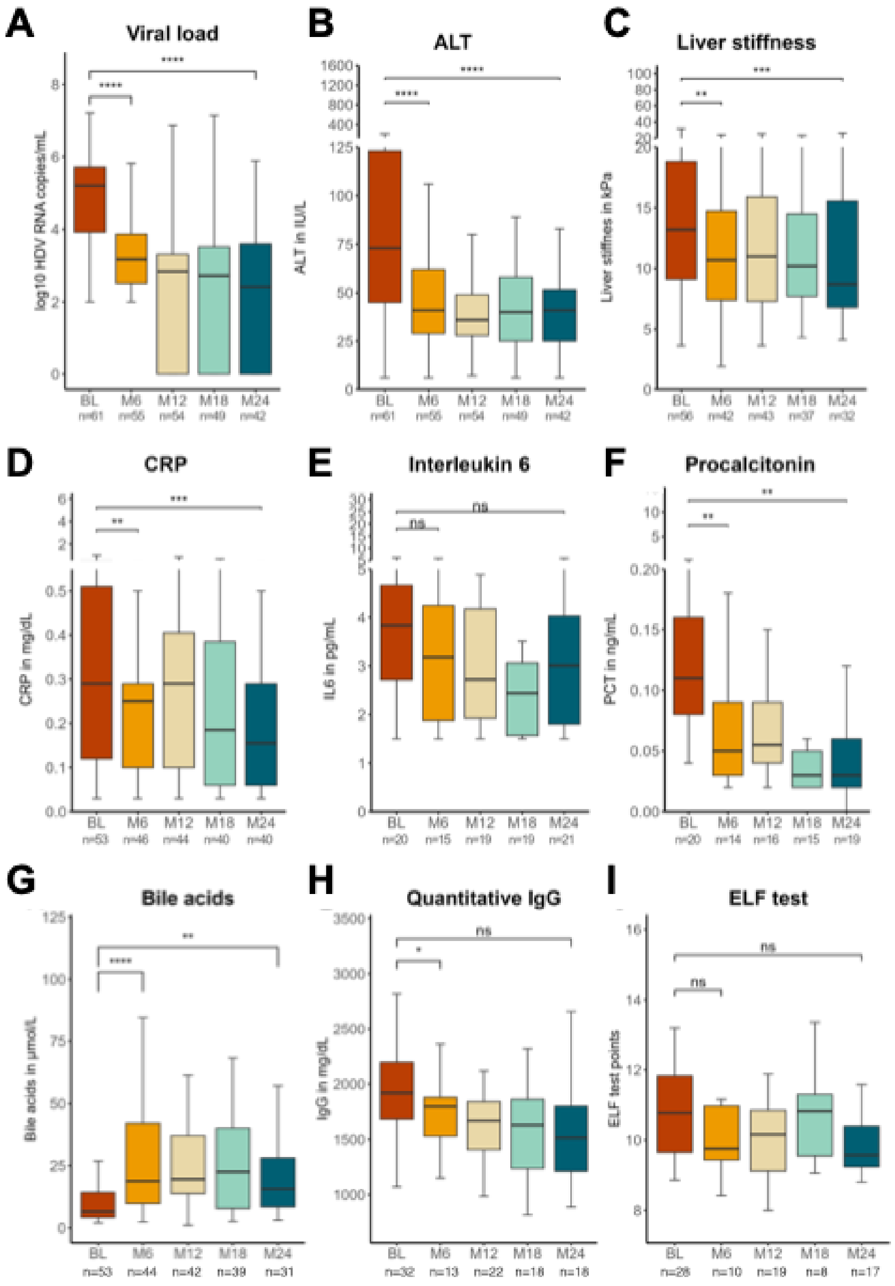

Fig. S2. Effects of BLV treatment on HDV-RNA, ALT levels, liver stiffness, CRP, IL-6, PCT, BA, quantitative IgG and the ELF test after 6, 12, 18, and 24 months of treatment. Bile acids increased after treatment initiation but then stabilized or regressed. Quantitative IgG decreased significantly with treatment duration as did the ELF test points, although not statistically significant.

Abbreviations: BL, baseline; ALT, alanine aminotransferase; CRP, C-reactive protein; ELF, enhanced liver fibrosis; HDV, hepatitis D virus; IgG, immunoglobulin G; IL6, interleukin 6; M, month, PCT, procalcitonin; RNA, ribonucleic acid.

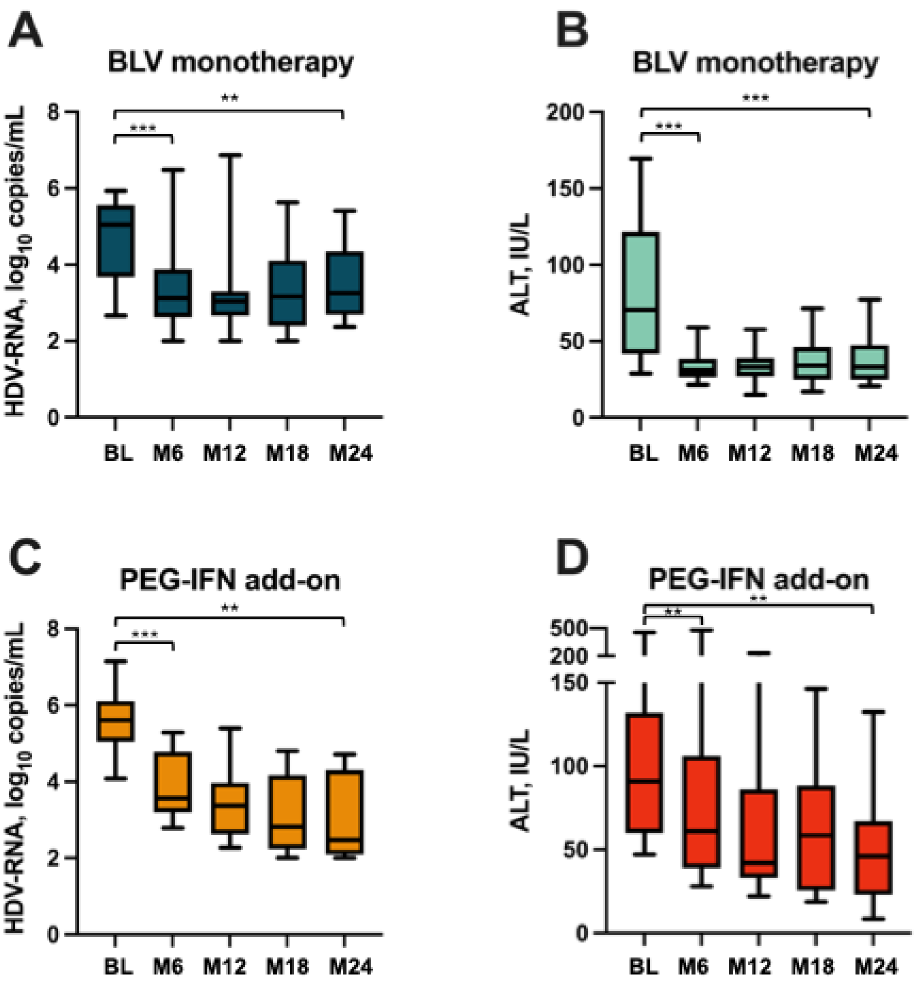

Fig. S3. Dynamics of HDV-RNA and ALT levels in patients with (A, B) BLV monotherapy (n=42) compared to those (C, D) with BLV+PEG-IFN add-on therapy (n=19). Of a total of 61 patients, 42 received BLV monotherapy throughout the observation period, whereas 19 patients were administered PEG-IFN as an add-on at some point during their BLV treatment course. (A, B) illustrating HDV-RNA (A) and ALT (B) trajectories in patients treated exclusively with BLV monotherapy. (C, D) depicting the corresponding biomarker dynamics in patients who received PEG-IFN add-on therapy. Biomarker levels are displayed at baseline and at 6-month intervals following initiation of BLV therapy (M6, M12, M18, M24). All measurements are aligned solely to BLV treatment time points. p values were mapped as “ns” for “not significant”, “*” for “p<0.05”, “**” for “p<0.01”, “***” for “p<.001”, and “****” for “p<0.0001”.

Abbreviations: ALT, alanine aminotransferase; BL, baseline; BLV, bulevirtide; HDV, hepatitis D virus; M, month; PEG-IFN, pegylated interferon alfa-2a; RNA, ribonucleic acid

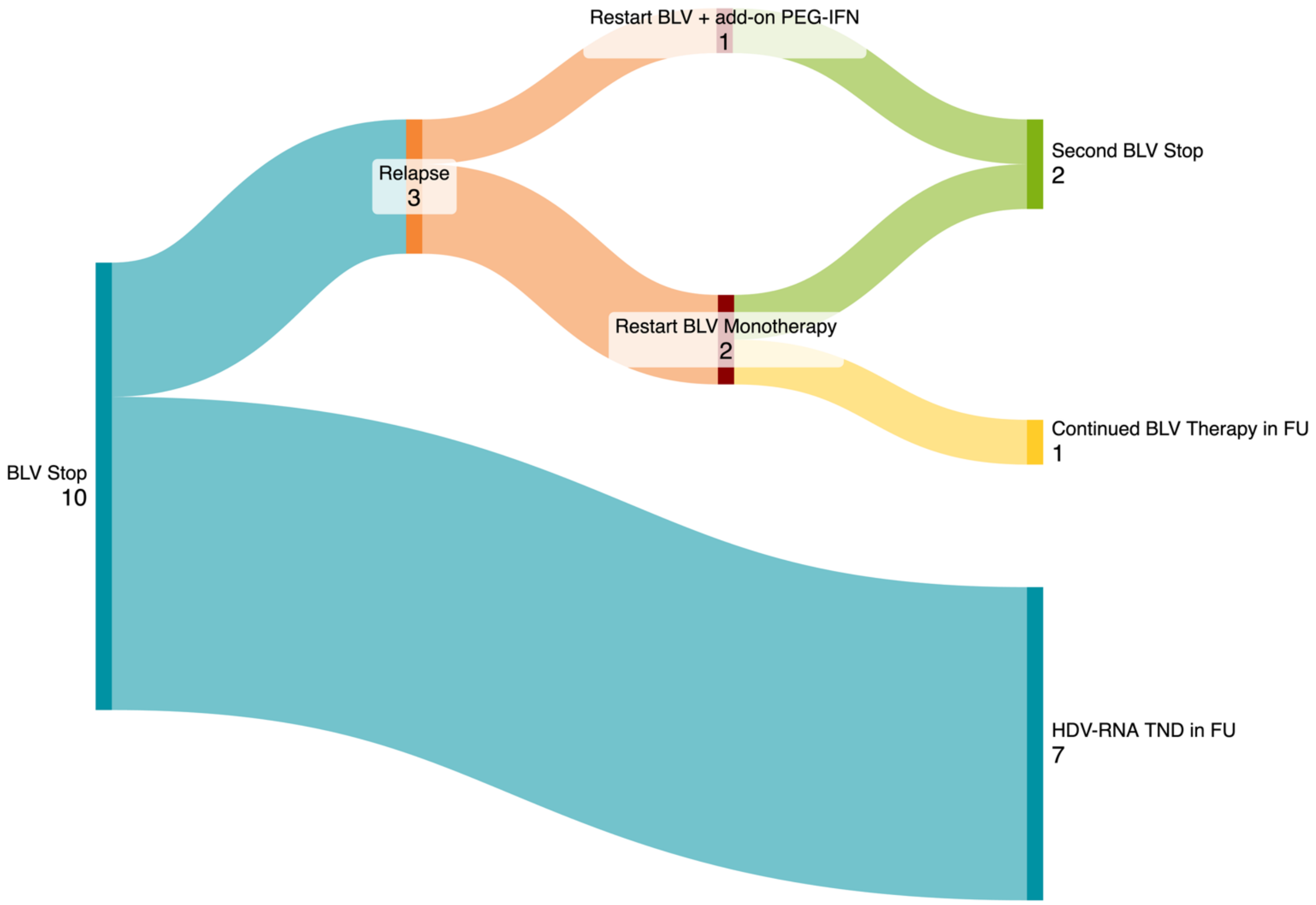

Fig. S4. Course of patients with BLV treatment discontinuation.

Abbreviations: BLV, bulevirtide; HDV-RNA, hepatitis D virus ribonucleic acid; FU, follow up; PEG-IFN, pegylated interferon alfa-2a; TND, target not detected.

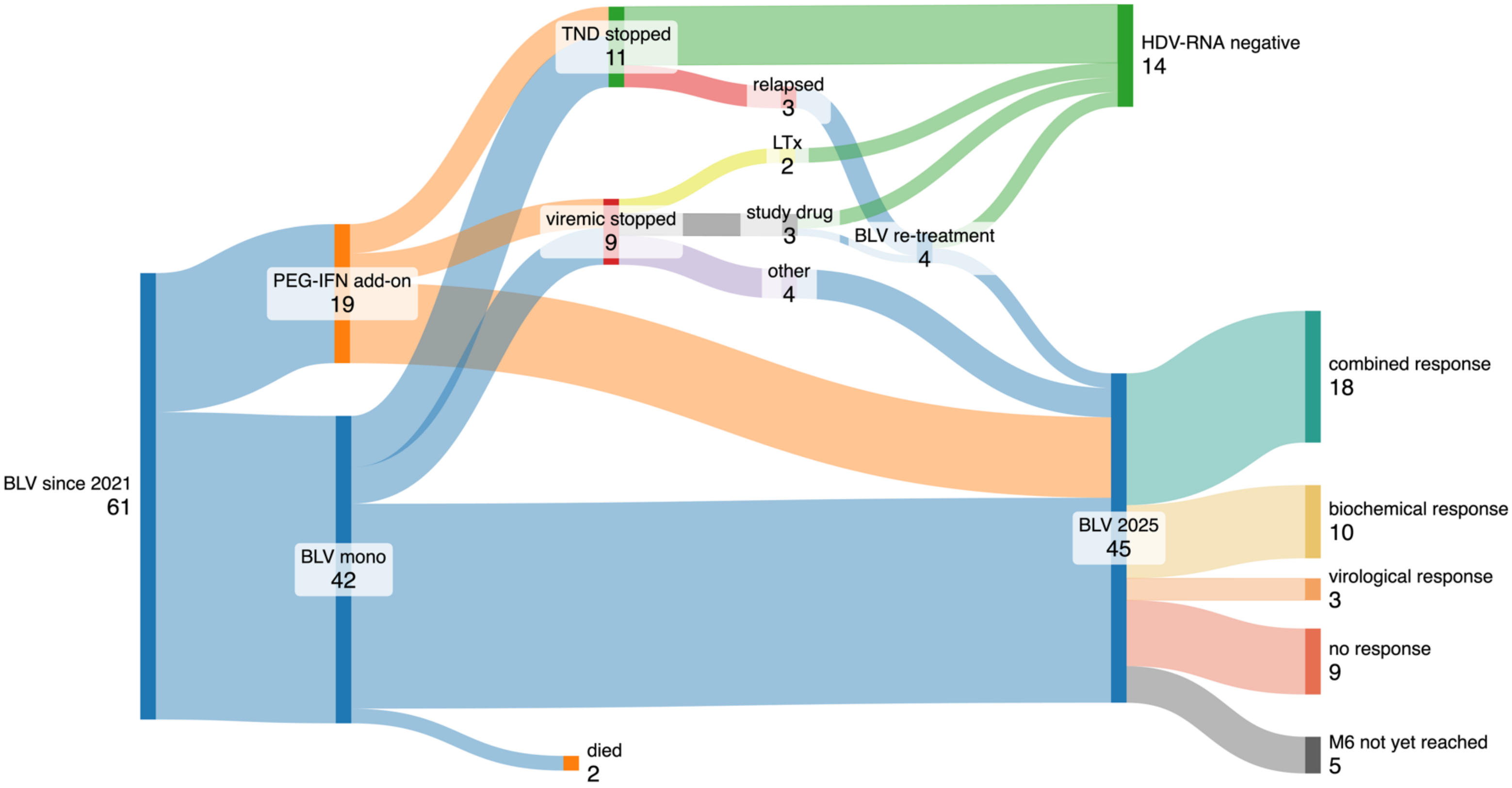

Fig. S5. Course of treatment of patients receiving BLV in Austria. Of a total of 61 patients, 19 (31.1%) received BLV with add-on IFN treatment. Ten patients achieved HDV-RNA TND and were electively discontinued. Of these patients, 3 had received add-on PEG-IFN. Three patients with HDV-RNA TND showed virological relapse, all of which had not received add-on PEG-IFN before, and were subsequently restarted on BLV. Some patients who did not achieve TND stopped BLV treatment for other reasons (allergy, pruritus, insurance issues, wish for children) and were later re-initiated. Of the 46 patients on active BLV treatment in 2025, 5 have not reached their M6 evaluation. Of the remaining patients, 43.9% achieved combined response, 70.7% biochemical response, and 51.2% virological response at the latest evaluation. In the entire cohort, two patients received liver transplantation without HDV relapse and two patients died on BLV treatment.

Abbreviations: BLV, bulevirtide; HDV, hepatitis D virus; IFN, interferon; LTx, liver transplantation; M, month; mono, monotherapy; PEG-IFN, pegylated interferon alfa-2a; RNA, ribonucleic acid; TND, target not detected.
