# Supplementary material for: Response-guided bulevirtide ± pegylated interferon alfa-2a: Long-term outcomes observed in the nationwide Austrian hepatitis D cohort study
Source: JHEP Rep. 2026 Mar 26;8(6):101835. doi: 10.1016/j.jhepr.2026.101835 (PMC13199667; doi:10.1016/j.jhepr.2026.101835)
Supplement: Multimedia component 2 [file mmc2.docx]

**JHEP Reports**

**CTAT methods**

Tables for a “Complete, Transparent, Accurate and Timely account” (CTAT) are now mandatory for all revised submissions. The aim is to enhance the reproducibility of methods.

- Only include the parts relevant to your study
- Refer to the CTAT in the main text as ‘Supplementary CTAT Table’
- Do not add subheadings
- Add as many rows as needed to include all information
- Only include one item per row

**If the CTAT form is not relevant to your study, please outline the reasons why:**

|  |
| --- |

- 1. **Antibodies**

| **Name** | **Citation** | **Supplier** | **Cat no.** | **Clone no.** |
| --- | --- | --- | --- | --- |
| **NA** |  |  |  |  |

- 1. **Cell lines**

| **Name** | **Citation** | **Supplier** | **Cat no.** | **Passage no.** | **Authentication test method** |
| --- | --- | --- | --- | --- | --- |
| **NA** |  |  |  |  |  |

- 1. **Organisms**

| **Name** | **Citation** | **Supplier** | **Strain** | **Sex** | **Age** | **Overall n number** |
| --- | --- | --- | --- | --- | --- | --- |
| **NA** |  |  |  |  |  |  |

- 1. **Sequence based reagents**

| **Name** | **Sequence** | **Supplier** |
| --- | --- | --- |
| **NA** |  |  |

- 1. **Biological samples**

| **Description** | **Source** | **Identifier** |
| --- | --- | --- |
| **NA** |  |  |

- 1. **Deposited data**

| **Name of repository** | **Identifier** | **Link** |
| --- | --- | --- |
| **NA** |  |  |

- 1. **Software**

| **Software name** | **Manufacturer** | **Version** |
| --- | --- | --- |
| Microsof Excel | Microsoft, Redmond, WA, USA | Office 2019 |
| Rstudio | Posit Software, Boston, MA, USA | Build 764 |

- 1. **Other (*e.g*. drugs, proteins, vectors etc.)**

| **Bulevirtide** |  |  |
| --- | --- | --- |
| **PEG-IFN** |  |  |

- 1. **Please provide the details of the corresponding methods author for the manuscript:**

| Prof. Thomas Reiberger, MD  Division of Gastroenterology and Hepatology  Department of Medicine III  Medical University of Vienna  Waehringer Guertel 18-20  A-1090 Vienna, Austria  [thomas.reiberger@medunwien.ac.at](mailto:thomas.reiberger@medunwien.ac.at)  Tel/Fax: +43 1 40400-47500 / +43 1 40400-47350 |
| --- |

**2.0 Please confirm for randomised controlled trials all versions of the clinical protocol are included in the submission. These will be published online as supplementary information.**

| **NA** |
| --- |
